# Does Disruption of Optic Atrophy-1 (OPA1) Contribute to Cell Death in HL-1 Cardiomyocytes Subjected to Lethal Ischemia-Reperfusion Injury?

**DOI:** 10.3390/cells11193083

**Published:** 2022-09-30

**Authors:** Andrew R. Kulek, Vishnu V. R. Undyala, Anthony R. Anzell, Sarita Raghunayakula, Lee Ann MacMillan-Crow, Thomas H. Sanderson, Karin Przyklenk

**Affiliations:** 1 Department of Biochemistry, Microbiology and Immunology, Wayne State University School of Medicine, Detroit, MI 48201, USA; 2 Department of Physiology, Wayne State University School of Medicine, Detroit, MI 48201, USA; 3 Clinical Research Institute, Children’s Hospital of Michigan & Department of Pediatrics, Central Michigan University, Detroit, MI 48201, USA; 4 Department of Human Genetics, School of Public Health, University of Pittsburgh, Pittsburgh, PA 15213, USA; 5 Department of Emergency Medicine, Michigan Medicine, University of Michigan, Ann Arbor, MI 48109, USA; 6 Department of Pharmacology & Toxicology, University of Arkansas for Medical Sciences, Little Rock, AR 72205, USA; 7 Departments of Physiology and Emergency Medicine, Wayne State University School of Medicine, Detroit, MI 48201, USA

**Keywords:** ischemia-reperfusion injury, cardiomyocyte, ischemic preconditioning, remote ischemic conditioning, mitochondrial morphosis, OPA1, OMA1 cristae remodeling, apoptosis

## Abstract

Disruption of mitochondrial structure/function is well-recognized to be a determinant of cell death in cardiomyocytes subjected to lethal episodes of ischemia-reperfusion (IR). However, the precise mitochondrial event(s) that precipitate lethal IR injury remain incompletely resolved. Using the in vitro HL-1 cardiomyocyte model, our aims were to establish whether: (1) proteolytic processing of optic atrophy protein-1 (OPA1), the inner mitochondrial membrane protein responsible for maintaining cristae junction integrity, plays a causal, mechanistic role in determining cardiomyocyte fate in cells subjected to lethal IR injury; and (2) preservation of OPA1 may contribute to the well-documented cardioprotection achieved with ischemic preconditioning (IPC) and remote ischemic conditioning. We report that HL-1 cells subjected to 2.5 h of simulated ischemia displayed increased activity of OMA1 (the metalloprotease responsible for proteolytic processing of OPA1) during the initial 45 min following reoxygenation. This was accompanied by processing of mitochondrial OPA1 (i.e., cleavage to yield short-OPA1 peptides) and release of short-OPA1 into the cytosol. However, siRNA-mediated knockdown of OPA1 content did not exacerbate lethal IR injury, and did not attenuate the cardioprotection seen with IPC and a remote preconditioning stimulus, achieved by transfer of ‘reperfusate’ medium (TRM-IPC) in this cell culture model. Taken together, our results do not support the concept that maintenance of OPA1 integrity plays a mechanistic role in determining cell fate in the HL-1 cardiomyocyte model of lethal IR injury, or that preservation of OPA1 underlies the cardioprotection seen with ischemic conditioning.

## 1. Introduction

Under conditions of cellular stress, maintenance of mitochondrial integrity is well- recognized to be a crucial determinant of cardiomyocyte fate. Therefore, it is not surprising that mitochondria have been implicated to play a pivotal role in the pathogenesis of lethal myocardial ischemia-reperfusion (IR) injury, with long-standing attention focused on the role of reactive oxygen species, mitochondrial calcium overload, and opening of the mitochondrial permeability transition pore in IR-induced cardiomyocyte death [1,2,3,4]. More recently, investigation of IR injury has expanded beyond these ‘classical’ mechanisms, with growing interest in the concept that mitochondrial quality control processes (i.e., fission, fusion and mitophagy) may play a mechanistic role in determining cell viability [5,6,7,8,9]. 

Mitochondrial fission and fusion are dynamically balanced under conditions of homeostasis. However, when this equilibrium is disrupted, mitochondrial quality control is compromised, favoring mitochondrial fragmentation and the activation of mitochondrial injury pathways [5,7,8,10,11,12]. These events are regulated by a family of dynamin-related GTPase proteins: dynamin-related protein 1 (DRP1) and optic atrophy protein (OPA1) [5,7,13,14]. DRP1, termed the ‘master-regulator’ of mitochondrial fission, has historically been implicated as a harbinger of injury during IR, prompting investigation into DRP1 inhibition as a potential strategy for mitigating IR injury [8,9,12,15]. Recent data, however, have yielded mixed outcomes [13,14]. 

OPA1, the second molecular regulator of morphosis, is a complex mitochondrial inner-membrane fusion protein that contributes to the maintenance of cristae architecture, modifies mitochondrial metabolism, and tethers cytochrome c within the cristae [15,16]. OPA1 exists as five distinct, fusion-competent peptides (i.e., OPA1 isoforms) in cardiomyocytes, generated through alternative splicing of the OPA1 gene, and through proteolytic processing of two higher molecular weight long-OPA1 isoforms (L-OPA1) to yield three truncated OPA1 peptides (S-OPA1) [17,18,19]. Our understanding of OPA1 is largely derived from non-cardiac models, where mitochondrial injury has been associated with the proteolytic processing of OPA1 via a cognate “stress” metalloprotease OMA1 [20,21,22,23]. Although data obtained from cardiac models remains limited, current evidence suggests that proteolysis of OPA1 may contribute to ischemia-reperfusion injury. Specifically: (i) IR has been shown to initiate OPA1 processing [24], (ii) genetic depletion of myocardial OPA1 reportedly sensitizes cardiomyocytes to apoptosis and mitochondrial network fragmentation [18], and (iii) cardiomyocyte injury is purportedly exacerbated when OPA1 expression is decreased [18,24,25,26]. 

While these previous studies implicate OPA1 in lethal IR-induced cell death, a requisite role for proteolytic processing of OPA1 as a mechanistic determinant of cardiomyocyte fate has not been definitively established. Moreover, with the exception of one published study [27], the concept that preservation of OPA1 may be cardioprotective and, in particular, contribute to the well-described resistance to IR-induced cardiomyocyte death evoked by ischemic conditioning paradigms (i.e., pre-post- and remote conditioning) [28,29,30] have not been explored. Accordingly, using the HL-1 cardiomyocyte model of simulated ischemia-reperfusion, our aims were to: (i) confirm the efficacy of ischemic preconditioning (IPC) and a remote conditioning stimulus achieved by transfer of ‘reperfusate’ medium (TRM-IPC) in this cell culture model, (ii) determine the effect of simulated IR on OMA1 activity and the integrity and subcellular localization of OPA1, (iii) establish whether ischemic conditioning alters the proteolysis and subcellular localization of OPA1, and (iv) quantify the effect of OPA1 knockdown on both the subcellular distribution of OPA1 and on cardiomyocyte viability in cells subjected to simulated IR, IPC and TRM-IPC. 

## 2. Materials and Methods

All components of this study were performed using HL-1 cardiomyocytes, a model initially developed by Claycomb and colleagues that maintains a stable, contracting phenotype over the course of ~20 passages [31]. 

HL-1 cardiomyocytes were maintained using standard methods described previously by our group [12,32]. Briefly: Cells were cultured in complete growth medium (CCM) composed of Claycomb medium supplemented with 10% fetal bovine serum, 2 mM L-glutamine, 0.1 mM norepinephrine, and penicillin/streptomycin antibiotic (100 U/mL; 100 ug/mL: All components purchased from Sigma-Aldrich, Inc., St. Louis, MO, USA) and maintained at 37 °C under normoxic conditions (defined as 95% ambient air/5% CO_2_). Cells were seeded at an initial density of 140,000 cells/cm^2^ and grown to >90% confluence on 60 mm gelatin/fibronectin-coated tissue culture dishes. Simulated ischemia was induced by subjecting cells to oxygen-glucose deprivation, achieved by media exchange to an ischemia-mimetic buffer (125 mM NaCl, 8 mM KCl, 1.2 mM KH_2_PO_4_, 1.25 mM MgSO_4_, 1.2 mM CaCl_2_, 6.25 mM NaHCO_3_, 20 mM HEPES, 5.5 mM glucose, 20 mM 2-deoxyglucose, 5 mM Na-lactate: pH 6.6) together with incubation at 37 °C in a sealed hypoxic chamber with GasPak EZ Gas Generating Sachets (BD Biosciences, San Jose, CA, USA) [33,34,35]. Reoxygenation was established by media exchange to serum-free Claycomb medium (SFCCM) and restoration of normoxia. Normoxic groups were maintained for a time-matched period in SFCCM. 

### 2.1. Protocol 1: Ischemic Conditioning Attenuates Cardiomyocyte Death in the HL-1 Cell Model

**Figure 1:**
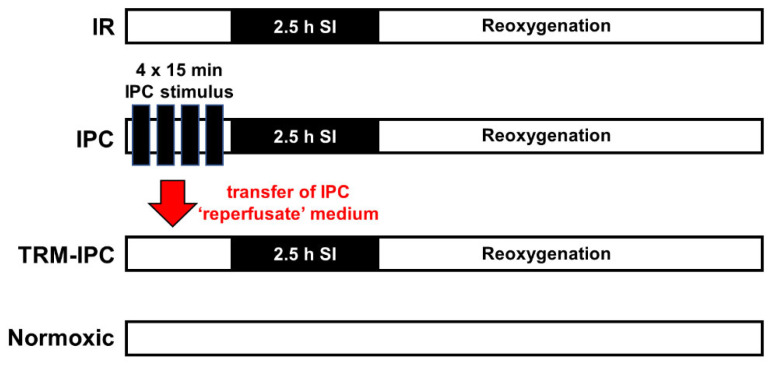
Timelines for IR, IPC, TRM-IPC and time-matched normoxic control groups. In Protocols 1 and 2A: HL-1 cells were cultured for 48 h before group assignments were made. Groups were reoxygenated for 24 h for the assessment of viability (Protocol 1), reoxygenated for 30 min for assessment of cytosolic expression of cytochrome c and cleaved caspase 3 (Protocol 1, Supplementary Material), or reoxygenated for 30 min for assessment of the subcellular distribution of OPA1 (Protocol 2A). In Protocol 3: The same 4 group assignments were made. However, for each group, (IR, IPC, TRM-IPC, normoxic control), three transfection cohorts were assessed: Cells were transfected with siRNA targeting OPA1, transfected with scrambled siRNA, or non-transfected. IR = simulated ischemia-reoxygenation, IPC = ischemic preconditioning, TRM-IPC = transfer of IPC ‘reperfusate’ medium.

Our aim in Protocol 1 was to confirm that protection against lethal simulated IR injury could be achieved in this in vitro cell culture model using a standard IPC stimulus, and establish that cytoprotection could be evoked by applying a remote conditioning strategy. To achieve this goal, HL-1 cells were cultured on 60 mm plates and assigned to undergo simulated IR alone, IPC + simulated IR, TRM-IPC + simulated IR, or a time-matched normoxic period (Figure 1). ***For the IPC-treated group***, cells were subjected to 4 episodes of [15 min simulated I + 15 min R] (algorithm optimized in preliminary studies; data not shown). Immediately following the fourth episode of IPC, the cardiomyocytes were exposed to a sustained, 2.5 h period of simulated I followed by 24 h of R. ***For the TRM-IPC-treated group:*** the ‘reperfusate’ medium was collected during each reoxygenation period from each dish of HL-1 cells subjected to IPC and, after the final IPC cycle, was transferred to a dish of naïve and normoxic HL-1 cardiomyocytes. Cells were treated for 1 h with the transferred, reperfusate medium and then underwent 2.5 h simulated I + 24 h R. ***For the IR group,*** Hl-1 cells underwent 2.5 h simulated I + 24 h R alone, while additional cultures served as time-matched **normoxic controls** (Figure 1).

The primary endpoint of Protocol 1 was HL-1 cell viability, assessed at 24 h post-R using the standard method of Trypan blue exclusion [12,36]. The method is based on maintenance of sarcolemmal integrity: viable cells with an intact sarcolemma remain unstained, while dead cells are permeable to the dye and appear blue. For each group, % cell viability was calculated as [1 − dead cells/(dead cells + live cells)] × 100. 

To ensure the rigor of our data, two sets of supplementary experiments were performed. First, HL-1 cell viability was confirmed with an additional technique, the well-established MTT ((3-(4,5-dimethylthiazol-2-yl)-2,5-diphenyltetrazolium bromide) assay (see Supplementary Figure S1) [37,38]. The protocol was identical to that shown in Figure 1, except the HL-1 cells were cultured in 24-well (rather than 60 mm) plates [12,32]. The MTT assay is based on the formation and accumulation of purple formazan crystals following reduction of the tetrazolium salt by cellular dehydrogenases present in viable cells [39]. Absorbance of each independent replicate at a wavelength of 570 nm, measured using a Bio-RAD iMark plate reader and Microplate Manager^®^ 6 Software (Bio-RAD, Hercules, CA, USA), was used to quantify formazan product formation and served as the index of viability. For each group, % cell viability was calculated as [A_570_ treatment group (i.e., IR, IPC or TRM-IPC)/A_570_ normoxic group] × 100. Second, HL-1 cells were probed by standard immunoblotting at 30 min post-R for two canonical markers of apoptotic cell death: the presence of cytochrome c and cleaved caspase 3 in the cytosol (primary antibodies from Cell Signaling Technologies, Boston, MA, USA; #11940 and #9664). Samples were loaded at 10–45 µg of protein (for cytochrome c and cleaved caspase 3, respectively) and resolved using methods described previously by our group [12,40].

### 2.2. Protocol 2

#### 2.2.1. Protocol 2A: Effect of Simulated Ischemia-Reperfusion Injury on the Integrity and Subcellular Localization of OPA1

Myocardial ischemia-reperfusion injury is reportedly associated with the proteolytic processing and loss of the key inner membrane fusion protein OPA1 [24,26,41,42,43]. Accordingly, our aims in Protocol 2A were to: (i) establish whether simulated IR in the HL-1 cardiomyocyte model initiates proteolytic processing of OPA1 and subcellular redistribution of OPA1 from mitochondria to the cytosol; and (ii) determine whether IPC and TRM-IPC attenuate the IR-associated processing of mitochondrial OPA1. 

To investigate these concepts, HL-1 cells were cultured and maintained as described in Protocol 1 and assigned to undergo IPC, TRM-IPC, simulated IR alone, or a time-matched normoxic period. At 30 min post-reoxygenation (a timepoint guided by published data from our group [12] and based on preliminary experiments), cells were harvested and interrogated by Western blot analysis for whole-cell and subcellular changes in OPA1. 

For evaluating changes in total cellular OPA1 content, cell lysates were prepared from HL-1 cells by detergent-based chemical lysis using ice-cold 1× RIPA lysis buffer (Pierce-Thermo Fisher Scientific Inc., Rockford, IL, USA) supplemented with 2× protease inhibitor (Roche Diagnostics, Indianapolis, IN, USA) and 2× phosphatase inhibitor (Pierce-Thermo Fisher Scientific Inc., Rockford, IL, USA). Lysates were gently agitated for 30 min at 4 °C and nuclei were pelleted by centrifugation at 12,000 rpm for 10 min at 4 °C; the supernatant was then collected, and protein concentration was quantified using the Bradford assay.

For subcellular fractionation into mitochondrial and cytosolic components, cells were harvested in an osmotically balanced, sucrose-mannitol homogenization buffer composed of: 10 mM HEPES pH 7.5, 1 mM EDTA and 1 mM EGTA (Boston BioProducts, Ashland, MA, USA), 10 mM KCl, 210 mM mannitol, 70 mM sucrose (Sigma-Aldrich, St. Louis, MO, USA), 1.5× protease inhibitor and 1.5× phosphatase inhibitor [44]. Mechanical lysis was achieved via ~50 passages through a 26G × 1/2” needle [12], and the resultant homogenates were initially centrifuged at 1000× *g* for 5 min at 4 °C to pellet cellular debris. The pellets were discarded, and the supernatants were centrifuged at 15,000 rpm for 20 min at 4 °C. The supernatant from this second centrifugation yielded the cytosolic fraction, while the remaining crude mitochondrial pellet was resuspended in homogenization buffer and centrifuged at 150,000× *g* for 10 min to obtain the mitochondrial-enriched membrane fraction. Homogenization buffer containing 1% Triton X-100 was used to solubilize mitochondrial membranes. Protein concentration was determined using the Bradford assay.

The endpoint of Protocol 2A was assessment of total OPA1, long OPA1 and short OPA1 isoform expression in whole cell lysates and subcellular fractions. Protein samples (loaded at 5–20 µg protein per lane) were resolved by SDS-PAGE and transferred to nitrocellulose membranes. Membranes were blocked in TBST (Tris-buffered saline (TBS) and 0.1% Tween-20) containing 5% blotting grade blocker (Bio-Rad Laboratories, Hercules, CA, USA) for 1 h. The OPA1 antibody (BD Biosciences, San Jose, CA, USA: #612606) was diluted in 5% BSA/TBST and blots were incubated overnight at 4 °C with gentle agitation. Immunoreactive bands were probed with horseradish peroxidase (HRP)-conjugated secondary antibody dissolved in 5% blotting grade blocker/TBST for 1 h at room temperature and visualized using a Bio-Rad imaging system (Bio-Rad Laboratories, Ann Arbor, MI, USA) or X-ray film. Quantification was performed using NIH ImageJ software: Versions 1.50 and 1.51, https://imagej.nih.gov/ij/(accessed on 20 December 2021). Expression of OPA1 in whole cell lysates, in cytosolic fractions and in mitochondrial fractions was normalized to the expression of beta-actin (Cell Signaling Technology, Boston, MA, USA #4970), GAPDH (Sigma-Aldrich, St. Louis, MO, USA: #G8795) and VDAC (Cell Signaling Technology, Boston, MA, USA: #4866), respectively [12].

#### 2.2.2. Protocol 2B: Effect of Simulated Ischemia-Reperfusion on OMA1 Activity

Disruption of OPA1 under conditions of stress is under the control of the zinc metalloprotease OMA1 [45]. If, as proposed in Protocol 2A, lethal IR injury in the HL-1 cell model is associated with proteolytic processing of OPA1, this should be accompanied (and preceded) by an increase in the enzymatic activity of OMA1. Our aim in Protocol 2B was to test this concept using a novel, quantitative assay for OMA1 activity recently developed by Tobacyk and colleagues [41].

To accomplish this, HL-1 cells underwent 2.5 h simulated I + 15 min R or a matched normoxic period. At 15 min post-R, cells were harvested and underwent detergent-based chemical lysis as described in Protocol 2A. In this proof-of-principle experiment, analysis was limited to normoxic and IR groups; IPC and TRM-IPC were not assessed.

The primary endpoint of Protocol 2B was the difference in OMA1 protease activity between normoxic and simulated IR groups, assayed using a fluorescent reporter substrate containing the eight amino acid OMA1 S1 cleavage site linked to an N-terminal 7-Methoxycoumarin-4-ylacetyl fluorophore moiety and C-terminal 2,4 Dinitrophenyl quencher moiety (generously donated to our lab by Dr. MacMillan-Crow; described in [41]). Using black, opaque 96-well plates (Costar-Corning, Midland, MI, USA #3792), 5 µg protein from HL-1 cell lysates was combined with the following reagents for a final total working volume of 100 µL per well: OMA1 activity assay buffer (50 mM Tris-HCl, pH 7.5, 40 mM KCl), +/−200 µM of the zinc chelator N,N,N′N′-Tetrakis (2-pyridylmethyl) ethylenediamine (TPEN, 1:250 dilution of stock TPEN dissolved in ethanol), and 5 µM final working concentration of OPA1 fluorescent reporter substrate (1:200 stock dissolved in DMSO). Three independent sets of normoxic cells and cells subjected to simulated IR were prepared, and, for each set, the assay was performed in triplicate at 37 °C on the same 96-well plate. Fluorescence was quantified at 5 min intervals, over a total duration of 30 min and corresponding to 15–45 min post-reoxygenation, using excitation and emission wavelengths of 325 nm and 405 nm, respectively. Corrected sample fluorescence was calculated by subtracting the intrinsic fluorescence of the OPA1 fluorogenic substrate alone from all sample fluorescence values. OMA1 activity was calculated as the difference in sample fluorescence with and without the addition of the zinc chelator TPEN, which inhibits the activity of OMA1 and therefore defines a near-zero-activity fluorescence value [41].

### 2.3. Protocol 3: Effect of OPA1 Knockdown on Cardiomyocyte Fate

Finally, if the processing of OPA1 seen in HL-1 cardiomyocytes subjected to simulated IR plays a causal, mechanistic role in lethal ischemia-reperfusion injury, we posited that genetic knockdown of OPA1 would exacerbate IR-induced cell death and/or attenuate the protection achieved with ischemic conditioning. Accordingly, our goal in Protocol 3 was to assess the effect of siRNA-mediated knockdown of OPA1 on HL-1 cardiomyocyte viability in IPC- and TRM-IPC-treated cells and cells subjected to IR alone.

To investigate this question, HL-1 cardiomyocytes were seeded onto 24-well plates for viability analysis by the MTT assay (see Protocol 1, supplementary experiments) or 60 mm tissue culture dishes for Western blot analysis (see Protocol 2A). After equilibration, cells were transfected as per the manufacturer’s protocol and as described by Dong et al. [12] using DharmaFECT 1 transfection reagent (1.25 µg/uL: GE Healthcare Dharmacon, Lafayette, CO, USA; #T-2001-03) and either siRNA targeting OPA1 (50 nM) or non-targeting (scrambled) siRNA (50 nM) in antibiotic-free CCM (GE Healthcare Dharmacon, Lafayette, CO, USA; #L-042427-01-0005 and #D-001206-13-05, respectively). Reagent concentrations were chosen based on preliminary data showing that >80% knockdown of OPA1 expression with minimal cytotoxicity was achieved with 50 nM of OPA1 siRNA and 1.25 µg/µL transfection reagent (see Supplementary Figure S3).

Cohorts of non-transfected HL-1 cells, HL-1 cells transfected with OPA1 siRNA, and HL-1 cells transfected with scrambled siRNA were subjected to IPC, TRM-IPC, simulated IR alone, or a time-matched normoxic period as detailed in Protocol 1 and shown in Figure 1. The primary endpoint in Protocol 3 was cell death assessed by the MTT assay at 24 h post-R. 

### 2.4. Statistical Analysis

Statistical analysis was performed using GraphPad Prism software Version 9 (La Jolla, CA, USA). All data are normally distributed (determined using the Shapiro–Wilk test) and are presented as group means ± SD (standard deviation). One-way ANOVA (analysis of variance) or two-way ANOVA (for group and time) was utilized for analysis of three or more groups and, when significant *p*-values were obtained, pairwise post hoc comparisons were made using Tukey’s test. For comparisons of two groups, unpaired *t*-tests were performed. *p*-values < 0.05 were considered statistically significant. 

## 3. Results

### 3.1. Protocol 1

**Figure 2:**
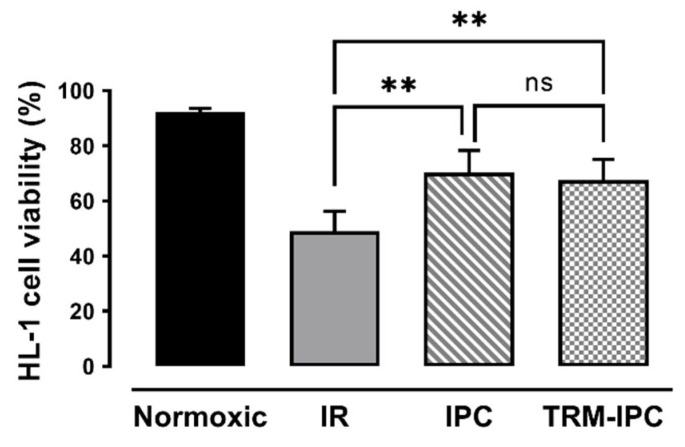
Protocol 1: Ischemic conditioning attenuates HL-1 cardiomyocyte death. HL-1 cell viability, assessed by Trypan blue staining, in IPC, TRM-IPC, simulated IR and normoxic groups. Both IPC and TRM-IPC were cytoprotective: ** *p* < 0.01 versus simulated IR; ns = not significant; *n* = 10 replicates per group. IR = simulated ischemia-reoxygenation, IPC = ischemic preconditioning, TRM-IPC = transfer of IPC ‘reperfusate’ medium.

**Figure 3:**
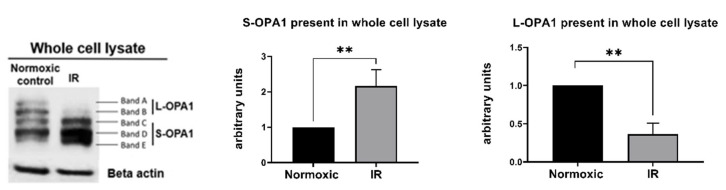
Protocol 2A: Simulated IR increases proteolytic processing of L-OPA1 to S-OPA1, with S-OPA1 redistribution to the cytosol. Original immunoblots (*left panel*) and quantified fold-changes (*right panels*) in OPA1 expression for HL-1 cells subjected to 2.5 h simulated I + 30 min R. Simulated IR was associated with the formation of S-OPA1 and the loss of L-OPA1 content in whole cell lysates (** *p* < 0.01 versus time-matched normoxic group). *n* = 5 replicates per group.

In time-matched normoxic cultures, HL-1 cell viability was maintained at >90%, while, in cells subjected to simulated IR, viability at 24 h post-reoxygenation was reduced to 49 ± 7% by Trypan blue exclusion assay (Figure 2). As expected, both IPC and TRM-IPC were cytoprotective versus the IR group (viability of 70 ± 8% and 68 ± 7%, respectively, *p* < 0.01), with no significant difference in efficacy between the two conditioning paradigms (Figure 3). These viability data were corroborated using the MTT assay (Supplementary Figure S1). In addition, and as expected, release of cytochrome c into the cytosol and expression of cleaved caspase 3 in the cytosol (considered harbingers of apoptotic cell death) were attenuated at 30 min post-R in IPC- and TRM-IPC-treated cohorts when compared with HL-1 cells subjected to IR alone (Supplementary Figure S2).

### 3.2. Protocol 2

OPA1 is a complex mitochondrial protein composed of 5 cleavage isoforms: 2 higher molecular weight long (L) forms and 3 lower molecular weight short (S) forms [40] (Figure 3). As expected [24,26,41,42,43], preliminary experiments revealed that HL-1 cells subjected to simulated IR displayed rapid proteolytic processing of OPA1 following reoxygenation, with loss of higher molecular weight L-OPA1 detected as early as 5 min post-R (data not shown).

**Figure 4:**
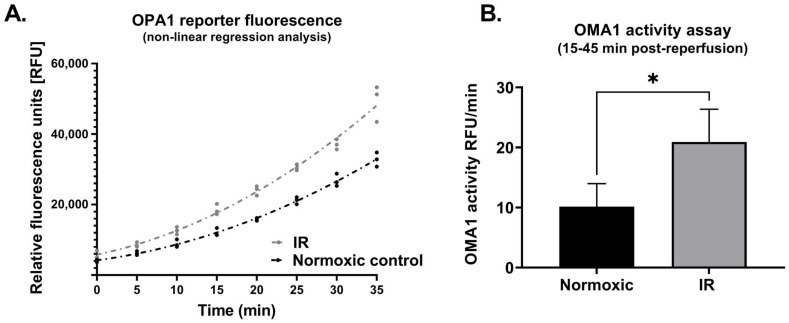
Protocol 2B: OMA1 protease activity is increased following reoxygenation. Panel (**A**): Non-linear regression analysis of OPA1 fluorogenic reporter peptide cleavage (assayed using a fluorescent reporter substrate: see Methods) in HL-1 cells subjected to simulated IR or a time-matched period of normoxia. Panel (**B**): OMA1 activity, assessed by quantifying initial reaction velocities (see Methods) was greater in cells subjected to simulated IR versus normoxia (* *p* < 0.05). *n* = 3 replicates per group. IR = simulated ischemia-reoxygenation.

In cells that underwent simulated IR, analysis of whole cell lysates revealed a significant reduction in expression of L-OPA1, together with an increase in S-OPA1 content (Protocol 2A; Figure 3). In addition, OMA1 protease activity, assessed at 15–45 min following reoxygenation, was significantly increased in cells subjected to simulated IR versus time-matched normoxic controls (Protocol 2B). This was reflected by the greater relative fluorescence at all time-points post-reoxygenation in HL-1 cells that underwent simulated IR (Figure 4A), and the greater value of OMA1 initial reaction velocity, defined as [Δ relative fluorescence units/Δ time] in the simulated IR group (Figure 4B).

**Figure 5:**
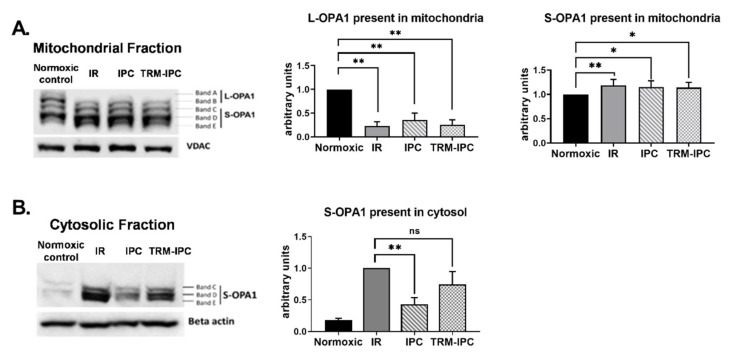
Protocol 2A: Effect of ischemic conditioning on OPA1 proteolysis and redistribution of S-OPA1. Original immunoblots (*left panels*) and quantified fold-changes (right panels) in expression of OPA1 mitochondrial and cytosolic fractions of HL-1 cells subjected to IPC, TRM-IPC, simulated IR or a matched period of normoxia. Panel (**A**). Significant processing of L-OPA1 was observed in IR, IPC and TRM-IPC groups versus normoxia (* *p* < 0.05; ** *p* < 0.01). Panel (**B**). Simulated IR was associated with release of S-OPA1 into the cytosol, an effect that was significantly attenuated by IPC (** *p* < 0.01) but not by TRM-IPC (ns = not significant). *n* = 5 replicates per group; normalized to beta-actin (cytosolic) or VDAC (mitochondria) loading controls. IR = simulated ischemia-reoxygenation, IPC = ischemic preconditioning, TRM-IPC = transfer of IPC ‘reperfusate’ medium.

Immunoblot analysis of subcellular fractions obtained from cells subjected to simulated IR demonstrated a robust decrease in expression of L-OPA1 in mitochondria (Figure 5A) and increased expression of S-OPA1 in the cytosol (Figure 5B). Release of S-OPA1 into the cytosol was significantly attenuated with IPC compared to the IR group but, in marked contrast, was not significantly reduced by TRM-IPC (Figure 5B). That is, although both conditioning strategies provided equivalent and significant cytoprotection (Figure 2), IPC and TRM-IPC had disparate effects on the release of processed OPA1 into the cytosol (Figure 5B). 

### 3.3. Protocol 3

In normoxic cells transfected with OPA1 siRNA (50 nM) for 48 h, the expression of OPA1 in whole cell lysates was decreased by >80% when compared with cells treated with either a non-targeting scrambled siRNA or non-transfected cells incubated in media alone (*p* < 0.01). This proved the most robust knockdown of all concentrations tested (see Supplementary Figure S3), with decreased expression of OPA1 seen in both the mitochondrial fractions (data not shown). Importantly, cytotoxicity associated with transfection was minimal: i.e., under normoxic conditions, viability assessed by the MTT assay was maintained at ≥90% of non-transfected control values in cells transfected with OPA1 siRNA or with scrambled siRNA (Supplementary Figure S3).

**Figure 6:**
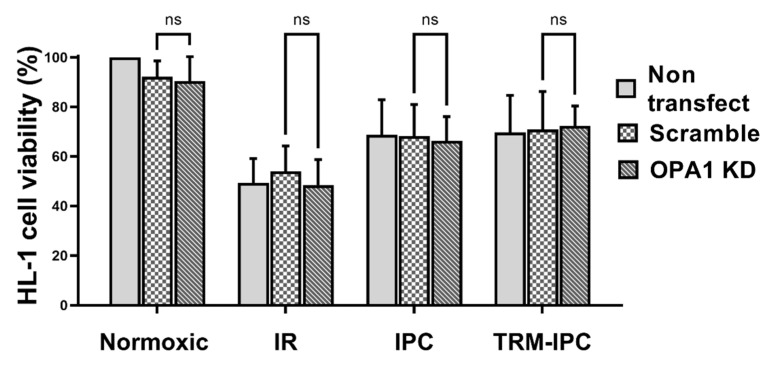
Protocol 3: Cell viability under conditions of OPA1 depletion. HL-1 cell viability, assessed by the MTT assay, in IPC, TRM-IPC, simulated IR and normoxic groups. For all groups, there were no differences in viability among non-transfected cells, cells transfected with scrambled siRNA and cells transfected with siRNA targeting OPA1 (ns = not significant). *n* = 5 replicates per group. IR = simulated ischemia-reoxygenation, IPC = ischemic preconditioning, TRM-IPC = transfer of IPC ‘reperfusate’ medium.

Finally, viability of all groups of HL-1 cells subjected to simulated IR alone was significantly reduced when compared with normoxic controls (Figure 6). siRNA-induced knockdown of OPA1 had no effect on viability of cells maintained under normoxic conditions, on the viability of cells that underwent simulated IR alone, or on the cytoprotective effects of IPC or TRM-IPC (Figure 6). Collectively, and contrary to our hypothesis, knockdown of OPA1 did not exacerbate simulated IR-induced HL-1 cell death, nor did it attenuate the efficacy of ischemic conditioning.

## 4. Discussion

In the current study, we provide evidence that, in HL-1 cardiomyocytes, simulated ischemia-reperfusion injury is accompanied by increased OMA1 protease activity, the proteolytic processing of mitochondrial OPA1 from L-OPA1 to S-OPA1 isoforms, and redistribution of S-OPA1 from mitochondria to the cytosol following reoxygenation. Most notably, our results fail to support the concept that maintenance of OPA1 integrity plays a mechanistic role in determining cell fate in the setting of simulated ischemia-reperfusion, or that preservation of OPA1 underlies the cardioprotection achieved with ischemic conditioning. Rather, our data suggest that, while proteolysis of OPA1 and redistribution of S-OPA1 to the cytosol occur in response to lethal IR injury, the acute depletion of cardiomyocyte OPA1 content (through siRNA knockdown) does not sensitize cardiomyocytes to lethal IR injury, nor does it attenuate the protective effect of IPC and TRM-IPC.

### 4.1. Ischemic Conditioning in Cell Culture Models

Ischemic conditioning (including the paradigms of IPC, remote ischemic preconditioning and postconditoning) is the well-described phenomenon whereby brief non-lethal periods of ischemia, applied locally or in a distant tissue or organ, render cardiomyocytes resistant to a more prolonged, lethal ischemic insult [28,30]. It is therefore not surprising that, in our in vitro model of ischemic conditioning, IPC and TRM-IPC were protective: i.e., both interventions improved viability following simulated ischemia-reperfusion. There is previous evidence that IPC can render cardiomyocytes resistant to a sustained period of simulated ischemia in cell culture models [28,46,47,48,49,50], and our results in Protocol 1 corroborate these observations. In contrast, in previous in vitro models of remote ischemic preconditioning, the remote stimulus was generated in vivo by limb ischemia applied to rodents or human subjects and transferred exogenously via serum or plasma to cultured cardiomyocytes [32,51]. Although this model does not fully replicate the complexities of a remote ischemic conditioning stimulus initiated in vivo (i.e., incorporates only the humoral component(s) produced by cells subjected to brief episodes of IR, and not the reported neuronal facets, of the phenomenon), our data demonstrate the feasibility of conducting a remote conditioning protocol entirely in cell culture via transfer of ‘reperfusate’ medium, and the potential utility of this reductionist model in interrogating the molecular mechanisms of this conditioning paradigm.

### 4.2. Total OPA1 Content and Lethal Ischemia-Reperfusion Injury

The first insight into the potential role of OPA1 in the setting of myocardial ischemia-reperfusion was provided in 2009 by Chen and colleagues, who reported that total OPA1 protein expression is reduced following simulated ischemia-reperfusion injury in H9c2 myoblasts, and that genetic depletion of OPA1 sensitizes the cells to mitochondrial fragmentation, apoptosis, and ischemia-reperfusion injury [18]. These concepts were supported by a subsequent in vivo study by the same group, where heterozygous OPA1 mutant mice were reportedly more susceptible to ischemia-reperfusion injury [25]. 

Only a handful of studies have subsequently evaluated OPA1 in cardiac models of ischemia-reperfusion injury, and discrepant results have been obtained. For example, the concept of an association between OPA1 depletion and changes in indices of apoptosis has not been confirmed in all studies [25,26], and modest OPA1 overexpression has failed to attenuate apoptosis and improve viability following ischemia-reperfusion [18]. In addition, OPA1 knockdown was accompanied by mitochondrial injury and upregulation of apoptosis in non-ischemic controls, suggesting that OPA1 knockdown itself has deleterious consequences [18,25]. Our data obtained in Protocol 3 do not support the results of Chen et al. [18,25]: siRNA-induced knockdown of OPA1 did not cause cell death in normoxic cardiomyocytes and did not exacerbate the death of HL-1 cells subjected to simulated ischemia-reperfusion. 

### 4.3. Ischemia-Reperfusion and OPA Processing

Previous studies have focused largely on manipulation of total cellular OPA1 content as a tool for interrogating a potential role of OPA1 in ischemia-reperfusion injury. Genetically modified OPA1 models have been useful in elucidating the contribution of the protein in regulating mitochondrial network morphology and cytochrome c retention; however, these studies do not yield insight into the potential role of OPA1 processing as a key step in the IR injury pathway. 

In the HL-1 cardiomyocyte model, we show that, under normoxic conditions, OPA1 exists as 5 distinct isoforms. In addition, we corroborate published data reporting that ischemia-reperfusion is associated with increased OMA1 protease activity and OPA1 proteolysis [19]: i.e., following simulated IR, we found a decrease in expression of L-OPA, an increase in expression of S-OPA1, together with a redistribution of S-OPA1 from mitochondrial fractions to the cytosol. Evidence from non-myocardial models has suggested that release of processed OPA1 occurs concomitantly with release of cytochrome c under conditions of apoptosis [52,53], and that cytosolic OPA1 promotes a feed-forward and complete release of both OPA1 and cytochrome c from the mitochondria [54]. Accordingly, these latter data implicate a potential cause-and-effect relationship between proteolytic processing of OPA1 and cell fate.

### 4.4. OPA1 Content, OPA1 Processing, and Cardioprotection

Despite >3 decades of investigation, the cellular mechanisms of ischemic conditioning remain incompletely resolved [1,29], a gap in knowledge that may contribute to the lack of success in translating this well-documented cardioprotective paradigm to the clinical setting [40,55,56,57,58,59,60,61,62,63]. There is a substantial body of evidence in support of the concept that conditioning-induced cardioprotection is due in part to preservation mitochondrial integrity and, in particular, maintenance of the mitochondrial permeability transition pore in a closed configuration [2,3,4,64,65,66,67,68]. In addition, there is one report describing an association between classic cardioprotective signaling pathways and modulation of mitochondrial morphology, favoring fusion and elongation [69]. However, only one study published to date has explored the potential relationship between conditioning-induced cardioprotection and molecular regulators of mitochondrial morphosis (i.e., DRP1 and OPA1), and found that the reduction of infarct size achieved with remote ischemic preconditioning was associated with preservation of total OPA1 content [27]. However, this study provided no insight into potential changes in OPA1 processing in the setting of ischemic conditioning, or the mechanism by which increased OPA1 may yield protection. 

If preservation of total OPA1 content plays a causal role in conditioning-induced cardioprotection, then depletion of OPA1 would presumably attenuate or abrogate the favorable effects of ischemic conditioning. Our results obtained in Protocol 3 do not support this concept: knockdown of OPA1 did not abrogate the improved viability seen with IPC and TRM-IPC. The outcome of Protocol 3 further demonstrates that IPC and TRM-IPC do not suppress the proteolytic cleavage and processing of L-OPA1. Release of S-OPA1 into the cytosol was significantly attenuated in IPC-treated H-1 cells when compared with cells subjected to simulated IR alone—an effect that, interestingly, was not seen in the TRM-IPC-treated cohort despite comparable improvements in cell viability achieved with both conditioning paradigms. This dissociation in cytosolic S-OPA1 expression between the IPC and TRM-IPC cohorts suggests that an attenuation in the subcellular redistribution of S-OPA1 is not required to evoke cardioprotection.

### 4.5. Summary, Limitations and Future Directions

In summary, our data demonstrate that loss of OPA1 content and integrity do not play a mechanistic role in determining cell fate in HL-1 cardiomyocytes subjected to simulated ischemia-reperfusion. In addition, neither preservation of OPA1 content, nor attenuation in the release of S-OPA1 into the cytosol, are required to achieve the improved cell viability seen with ischemic conditioning in this reductionist model.

It is important to note that our study focused exclusively on OPA1 content and proteolysis. Our protocols do not consider the concept developed from non-cardiac models that OPA1 processing and redistribution to the cytosol may involve compromised mitochondrial outer membrane integrity and a potential involvement of DRP1 [70,71,72,73], and were not designed to address the involvement of other mechanisms, independent of OMA1- mediated proteolytic processing of OPA1, in lethal ischemia-reperfusion injury and the protection achieved with ischemic conditioning. Second, aside from preliminary time-course studies that were limited in scope (data not shown), OPA1 content and proteolytic processing were probed at a single time-point following reoxygenation; we did not assess potential dynamic temporal changes in OPA1 that may occur during the minutes-hours following simulated IR, and did not incorporate imaging of mitochondrial networks or mitochondrial cristae integrity in our analysis [12,74]. Finally, our conclusions are based on data obtained from an immortal cardiomyocyte cell line. Additional studies, executed using rigorous and robust criteria [75], will be required to corroborate our observations in primary cardiomyocytes, as well as in in vivo models of myocardial ischemia-reperfusion.

## Data Availability

The data presented in this study are available on request from the corresponding author.

## Supplementary Materials

The following supporting information can be downloaded at: https://www.mdpi.com/article/10.3390/cells11193083/s1, Figure S1: Protocol 1: Ischemic conditioning attenuates HL-1 cardiomyocyte death; Figure S2: Protocol 1: Ischemic conditioning attenuates indices of apoptosis in the HL-1 cell model; Figure S3: Protocol 3: Effect of siRNA-mediated OPA1 knockdown on OPA1 expression and cell viability.
