# Supplementary material for: Does Disruption of Optic Atrophy-1 (OPA1) Contribute to Cell Death in HL-1 Cardiomyocytes Subjected to Lethal Ischemia-Reperfusion Injury?
Source: Cells. 2022 Sep 30;11(19):3083. doi: 10.3390/cells11193083 (PMC9564372; doi:10.3390/cells11193083)
Supplement: Supplementary file 1 [file cells-11-03083-s001.zip › cells-1827513-supplementary.pdf]

cells

Supplementary Material

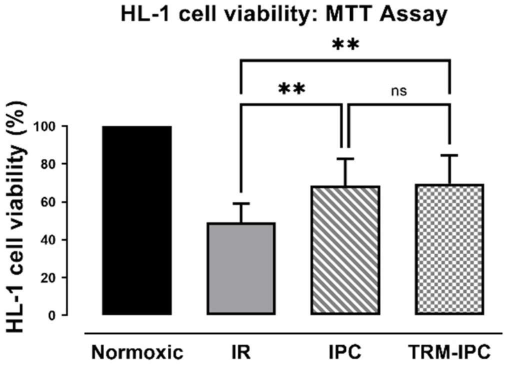

Figure S1. Protocol 1: Ischemic conditioning attenuates HL-1 cardiomyocyte death. HL-1 cell viability, assessed using the MTT assay, in IPC, TRM-IPC, simulated IR and normoxic groups. Both IPC and TRM-IPC were cytoprotective, corroborating the data obtained using Trypan blue staining (Figure 2): ** p <0.01 versus simulated IR; ns = not significant; n = 5 replicates per group; data normalized to normoxic control. IR = simulated ischemia-reoxygenation, IPC = ischemic preconditioning, TRM-IPC = transfer of IPC 'reperfusate' medium. preconditioning.

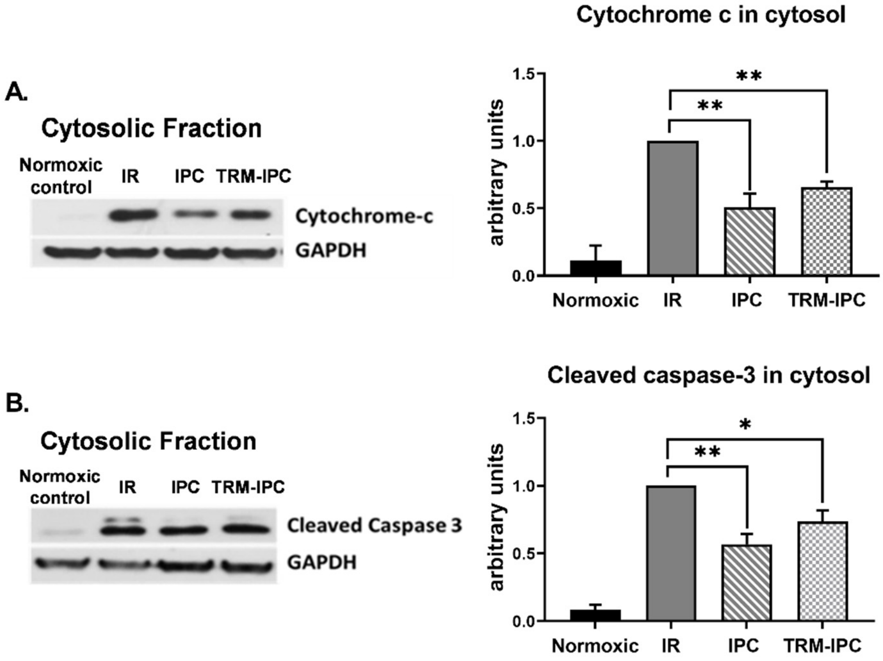

Figure S2. Protocol 1: Ischemic conditioning attenuates indices of apoptosis in the HL-1 cell model. HL-1 cells subjected to normoxia, IR alone, IPC+IR or TRM-IPC+IR were harvested and processed at 30 min post-R, and the cytosolic fractions were probed for expression of cytochrome c (Panel **A**: left) and cleaved caspase 3 (Panel **B**: left). Both IPC and TRM-IPC attenuated the IR-associated increase in cytosolic expression of cytochrome c (Panel **A**: right) and cleaved caspase 3 (Panel **B**: right): * p <0.05, ** p <0.01 versus IR; n = 3 replicates per group; expression normalized to GAPDH loading control. Abbreviations defined in Supplementary Figure S1.

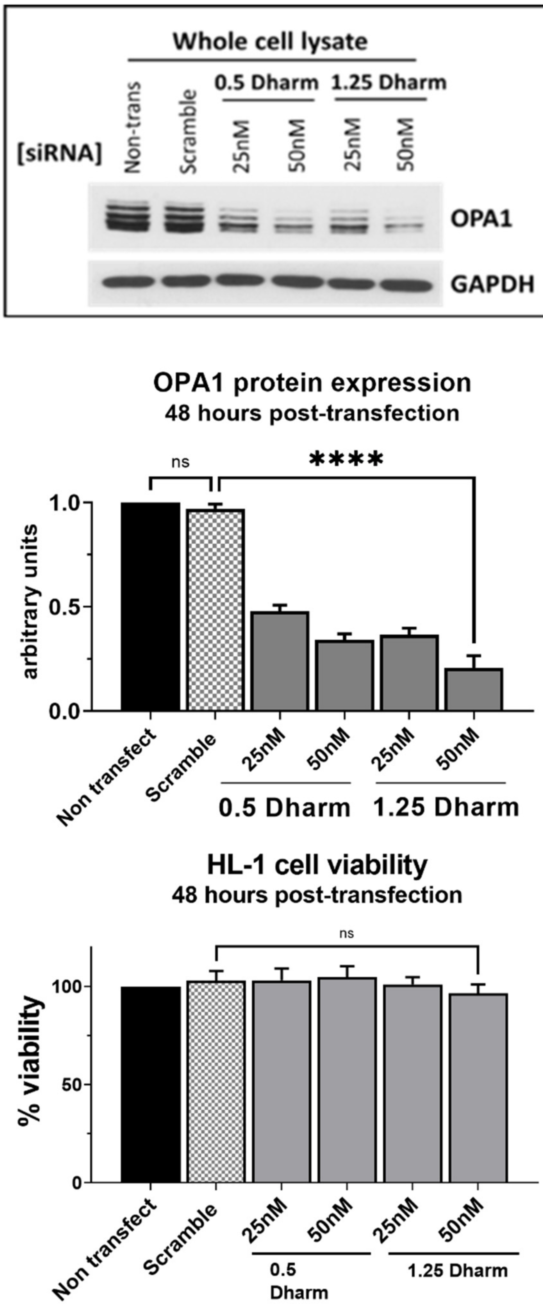

Figure S3. Protocol 3: Effect of siRNA-mediated OPA1 knockdown on OPA1 expression and cell viability. Top panel: Original immunoblots of whole cell lysates from HL-1 cells transfected for 48 h with scrambled siRNA, 25 nM or 50 nM siRNA targeting OPA1 and 0.5 μL/mL or 1.25 μL/mL Dharmafect transfection reagent. Middle panel: Quantified fold-changes in total OPA1 expression: **** p <0.01 versus scrambled siRNA; ns = not significant; n = 5 replicates per group; normalized to GAPDH. Bottom panel: HL-1 cell viability, assessed using the MTT assay, for cells transfected with scrambled siRNA or siRNA targeting OPA1. n = 3 replicates per group; Dharm = Dharmafect transfection reagent.
